# Dual pathways in social evolution: Population genetic structure of group-living and solitary species of kleptoparasitic spiders (Argyrodinae: Theridiidae)

**DOI:** 10.1371/journal.pone.0208123

**Published:** 2018-11-29

**Authors:** Yong-Chao Su, Po Peng, Mark Adrian Elgar, Deborah Roan Smith

**Affiliations:** 1 Department of Biomedical Science and Environment Biology / Graduate Institute of Medicine, Kaohsiung Medical University, Kaohsiung City, Taiwan; 2 Department of Ecology and Evolutionary Biology, University of Kansas, Lawrence, Kansas, United States of America; 3 School of BioSciences, University of Melbourne, Parkville, Victoria, Australia; University of Pretoria, SOUTH AFRICA

## Abstract

Group-living behavior is taxonomically widespread but rare in spiders. The conventional view is that the main pathways to group-living in spiders are either sub-social, where extended maternal care leads to prolonged sibling association; or communal living, where individuals aggregate to exploit a common resource. Female egg-sac guarding behavior occurs throughout kleptoparasitic spiders in the subfamily Argyrodinae (Theridiidae), while individuals in group-living species cohabit in the resource rich webs of their host spiders. These attributes fit both sub-social and communal routes to group-living, which offers new insights to study the early stages of social evolution. We investigated whether members of kleptoparasitic groups in natural populations comprise related individuals by comparing the population structure of two group-living species, *Argyrodes miniaceus* and *A*. cf. *fissifrons*, and two solitary species, *A*. *fasciatus* and *Neospintharus trigonum*. We found that: (1) genetic-spatial autocorrelation in group-living species was highest among spiders sharing the same host web and declined steeply with increasing distance, but no significant autocorrelation at any scale for solitary species; (2) there was high relatedness among group members in two cases of group-living species, which indicated relatedness was not an adhesive agent in most of the groups, but no high relatedness in solitary species; and (3) the host web boundary was not the sole predictor of genetic structures in group-living species. These results suggest that population genetic structure in the group-living species is caused by limited dispersal of group members that is favored by ecological conditions, including the nature and size of resources. In contrast, the absence of genetic structuring in populations of solitary species indicates a high level of dispersal with individual interactions unlikely to have fitness benefits.

## Introduction

Group-living has evolved in diverse animal taxa in response to a variety of environmental factors, including spatial distribution of resources [1], mating opportunities [2], risk of predation [3], parent-offspring aggregation [4], and group foraging [5]. The nature of the interactions among group members functions as the agent that increases the fitness of group members. These interactions are typically cooperative behaviors, ranging from reciprocal mutualism among non-related individuals to highly caste-differentiated eusocial groups [6, 7]. The conventional view is that the initial formation of groups may arise through sub-sociality, where sociality derives from extended maternal care and temporarily non-dispersed siblings, or through communal aggregations (typically comprising non-related individuals), where individuals aggregate to exploit a common resource [8–10]. Inclusive fitness theory [8, 11–13] helps explain the evolution of the initial transitioning stage of social evolution [14–16] (but see [12]), although empirical evaluations of these ideas are strongly biased toward particular taxa [17]. Most empirical studies of the early stages of social evolution involve species that have attributes that favor either the sub-social route, e.g., woodroaches [18] and Belding’s ground squirrels [9], or communal route, e.g., vampire bats [10].

Group-living is not widespread in spiders, primarily because this lifestyle must evolve from predatory, sometimes cannibalistic, solitary ancestors [19]. Nonetheless, group-living has evolved in several spider taxa [20–22]. Cooperative societies are characterized by extended maternal care, constraints on dispersal by the young, retention of mutual tolerance typical in newly hatched spiderlings, an inbred mating system, and cooperative behavior among colony-mates [19]. In contrast, communal groups originate from aggregations of individuals at valuable resources, such as nesting sites, web-building sites, or food resources [23, 24]. These groups typically exhibit extensive dispersal of immatures and little or no cooperation among group members. Groups may be ephemeral or permanent, and in some species obligate [23–25].

Fitness benefits from cooperation among sib groups have been investigated in several subsocial spiders. In the subsocial *Stegodyphus tentoriicola* (Eresidae), sib-groups were more likely to feed cooperatively; and in large groups more food was extracted from prey by siblings than non-siblings [26]. In the case of subsocial *S*. *lineatus* Schneider and Bilde [27] reared non-sib spiderlings with “foster mothers” to create groups comprised of unrelated but familiar spiderlings. Cooperation and feeding efficiency were higher in the sibgroups than in nonsibs, whether they were familiar with one another or not, demonstrating that relatedness–not mere familiarity–was the crucial factor for higher performace of sib-groups. However, Berger-Tal et al. [28] found no fitness benefits accrued to sibgroups when within-group relatedness was experimentally manipulated in the cooperative, permanently social *S*. *dumicola*. These studies suggest a potential role of kinship in the initial formation of subsocial groups.

However, these findings do not preclude a major role for ecological factors in the initial formation of groups [29]. Aggregation of individuals at patchily distributed resources can favor individuals exhibiting mutual tolerance, a requirement of group-living behavior. This dynamic has been proposed for colonial or orb-weaving species in the Araneidae (e.g., *Metepeira*, [30, 31]; *Cyrtophora citricola* [32]) and Uloboridae (e.g., *Philoponella oweni*, [23]) exploiting locally abundant food sources and patchy nesting substrates. These studies revealed how food resources influence the formation and maintenance of colonial groups. Relatedness among colonial group members is predicted to rise over time because some offspring remain at the resource, and because matings can take place among group members (e.g., *C*. *citricola*, [33]).

Social behavior among species of kleptoparasitic spiders of the subfamily Argyrodinae (Theridiidae) is unusual because their natural history seems to include both maternal care and a tendency to aggregate, attributes that fit both the sub-social and the communal routes to social behavior [34, 35]. Female egg-sac guarding behavior is ubiquitous in the Argyrodinae. At least some group-living Argyrodinae complete their life cycles on the host webs [36, 37]. Moreover, Argyrodinae are sister to a clade containing many subsocial and cooperative species that have extended maternal care [38]. Nevertheless, the origin of group-living in Argyrodinae may have also been influenced by the aggregation of individuals at rich resources–i.e., the host webs. The webs of host species may be considered as long-lasting, but still dynamic, habitat patches that provide stable resources for these kleptoparasites [37, 39, 40]. The duality of the evolutionary origins of group-living behavior in Argyrodinae contradicts the prevailing wisdom that social behaviour evolved through either maternal care or the formation of aggregations. While there are relatively few natural systems that allow observations of the transitions from solitary to social status [41], the group-living kleptoparasitic spiders in the subfamily Argyrodinae provide a unique opportunity to investigate the role of kinship and ecological conditions in the formation of sociality because there are several independent origins of group-living with this subfamily [35].

Applying genotyping methods to reveal patterns of relatedness of group members is a convenient way of understanding the role of kinship in the formation of social groups in natural populations. However, while genotyping methods are often used to test relatedness among individuals within natural groups of wasps and bees [42, 43], remarkably few studies use this technique for vertebrates (e.g., birds [44]), and rarely for spiders (but see [45–47]). We compare the population genetic structure of group-living species to that of solitary Argyrodinae species using amplified fragment length polymorphism (AFLP) data. We used the population genetic structure of solitary species as the baseline to test if the populations of group-living species are more genetically structured, and thus reveal whether kinship is a feature of group-living behavior in Argyrodinae. We assess which age and sex class of individuals are most likely to contribute to the genetic structure by investigating the population structure of juveniles, sub-adult and adult females, and sub-adult and adult males in group-living Argyrodinae. If groups of kleptoparasitic spiders on a single host web comprise mostly related individuals, then members of groups could benefit both directly and through inclusive fitness effects. However, inclusive fitness benefits would not accrue if the spiders that aggregate on a host are, on average, no more closely related than a random selection of individuals from the population.

## Material and methods

### Species selection

The population ecology of the kleptoparasitic Argyrodinae has been studied in nine of the 20 known group-living Argyrodinae species (reviewed in [48]), but only a single solitary species has been studied [49]. The major predictor of intra-specific kleptoparasite group size is the abundance of resources: group size is positively correlated with the availability of prey [40] and the web size [39, 50, 51]. Accordingly, larger resources predict larger groups of group-living Argyrodinae, a pattern that does not hold in the solitary species, *Neospintharus trigonum* [49].

There are two levels of spatial distribution in host-kleptoparasite systems: the distribution of the host, and the distribution of the kleptoparasites within host webs. Hosts may be clustered, uniformly distributed or randomly distributed. Host webs are the primary resource for the kleptoparasites, and group-living Argyrodinae are typically found in the larger webs of large-bodied spiders, such as *Nephila* (Araneidae) or *Cyrtophora* (Araneidae). Solitary Argyrodinae are often found in smaller host webs.

We follow Whitehouse [48] for definitions of group-living and solitary species. We examined the genetic structure of populations of two group-living species, *Argyrodes miniaceus* and *A*. cf. *fissifrons*, and two solitary species, *A*. *fasciatus* and *N*. *trigonum*, using DNA fingerprinting. These species are placed in different phylogenetic lineages within the Argyrodinae, each of which shows solitary kleptoparasitism as the ancestral condition [35], so the evolutionary contexts of sociality in these species are independent. Group-living *A*. *miniaceus* is a specialist kleptoparasite of *Nephila* hosts, and *A*. cf. *fissifrons* is a specialist kleptoparasite of *Cyrtophora* hosts. The solitary *A*. *fasciatus* is often found in the webs of sheet web spiders (Psechridae) [52], but are also found on the webs of *Cyrtophora*, *Nephila* and various Theridiidae. The solitary kleptoparasite *N*. *trigonum* is a generalist kleptoparasite that is found on the webs of many different clades of spiders, including *Argiope* (Araneidae), *Araneus* (Araneidae), *Agelenopsis* (Agelendiae), Linyphiidae, and Theridiidae.

### Population sample collection

Specimens of *A*. *miniaceus* were collected from the webs of *Nephila pilipes* in a single population in Huoyanshan, Taiwan (N 24° 21' 51", E 120° 44' 20”) in July 2007; *A*. cf. *fissifrons* from the webs of *C*. *moluccensis* in a single population in Lienhuachih, Taiwan (N 23° 54’ 51.1”, E 120° 53’ 17.3”) in December 2009; *A*. *fasciatus* from different hosts on Pulau Ubin Island, Singapore (N 1° 24' 24", E 103° 57' 58”) in December 2009; and *N*. *trigonum* from different hosts in Lawrence, KS (USA) (N 38° 56’ 58.2”, W 95° 16’ 7.3”) in 2011. All specimens were preserved in 95% ethanol for later identification of instar stages and DNA fingerprinting. We chose forest sites with a large number of host webs and made an exhaustive survey of all of the host webs along a transect (between 0.5 and 1 km long, and 50 m wide) until the habitat changed and no further host webs were found. The position of each web was determined using hand-held GPS (Garmin eTrex Summit HC, USA). We then converted the positions of the host webs from the geographic coordinates format (longitude and latitude) into Universal Transverse Mercator (UTM) coordinates for subsequent analyses. Each kleptoparasitic spider collected during surveys was allocated to one of five instar stages: first instar, spiderlings–legs I are not elongated; second instar–legs I are elongated, body size is close to that of spiderlings; third instar–slightly swollen palps in males, body size significantly larger than spiderling and second instar; fourth instar–genitalia of both sexes distinguishable but without detailed structures; and adult–with complete genitalia. The field research and collecting permit was issued by the National Parks Board, Singapore (NP/RP9800); non-national park areas in Taiwan and the campus of the University of Kansas, Lawrence, KS, USA do not require specific permits for arachnid research. No endangered or protected species was involved in this research.

### DNA extraction and fingerprinting

We extracted DNA from the whole specimen of spiderlings, second instar, or third instar spiders. We extracted DNA from the legs and cephorathorax of fourth instar and adult spiders, using Sigma-Aldrich DNA GeneElute kit (Sigma-Aldrich GN350, USA) following the commercial protocol for extraction of genomic DNA from each specimen.

We carried out DNA fingerprinting using the three-enzyme amplified fragment length polymorphism (TE-AFLP) method of van der Wurff et al. [53] (see S1 File, for detailed methodology of the TE-AFLP technique). We used *Xba*I, *Bam*HI *and Rsa*I to digest each DNA sample. The resulting DNA fragments were ligated to adaptors with ends complementary to the cut ends produced by *Xba*I and *Bam*HI. A subset of the DNA fragments was PCR amplified using primers complementary to the adaptors plus one to three additional arbitrary bases. We diluted PCR products and sized them in a Beckman CEQ 8000 automatic sequencer. The resulting data were imported to the program Genemapper *v* 4.0 (Thermo Fisher Scientific, USA) for scoring peak heights. We followed Whitlock *et al*. [54] to conduct peak height signal normalization, to call the phenotypes for each fragment, and to test the reliability of the data. We imported the raw peak height data that was screened in GeneMapper *v* 4.0 to AFLPscore 1.4b [54]. The total number of individuals on which we attempted TE-AFLP fingerprinting and the number of individuals successfully fingerprinted are shown in Table 1. The detailed descriptions of the TE-AFLP fingerprinting methods are in S1 File. The parameters used in the AFLP phenotype calling (0 = DNA fragment absent; 1 = DNA fragment present), the resulting number of individuals and loci retained in our data matrices, and the level of peak-calling mismatches between repeated samples are provided in Table 1. AFLP bands are dominant markers; individuals that are homozygous for presence of a DNA fragment (genotype 1, 1) cannot be distinguished from heterozygotes (1, 0) which inevitably reduces the power to discover informative loci [55]. Nevertheless, the AFLP method can compensate for this shortcoming, given its ability to detect a large number of polymorphic loci, in which it can efficiently identify individual profiles [55]. We retained the loci that have at least two individuals with present DNA fragments, i.e. at least two 1’s in those loci. The following population genetic analyses in group-living species do not include data where the webs contained one individual only.

**Table 1:** Results of mismatch rate test of repeatability between the original and repeated TE-AFLP data.

Species	*A*. *miniaceus*	*A*. cf. *fissifrons*	*A*. *fasciatus*	*N*. *trigonum*
**Mismatch rate (%)**	7.12	3.04	7.64	1.53
**Locus-selection threshold (rfu)**	800	80	300	120
**Phenotype calling threshold (rfu)**	100	400	100	100
**Normalized mean peak height (rfu)**	538.00	191.52	360.92	235.76
Retained loci*	191(221)	147 (208)	115 (131)	208 (262)
**Retained individuals/individuals sampled**	153/156	85/96	30/31	39/40

*The numbers in the parentheses are the retained loci from AFLP score [54]. We discarded all invariant loci and any locus for which only one individual presented a fragment or for which a fragment was present in all but one individual (singleton loci).

### Data analysis

#### Visualizing similarities of AFLP profiles using nonmetric multidimensional scaling (NMDS)

We generated pairwise Jaccard dissimilarity matrices using the AFLP phenotype tables, in which the index between two individuals was calculated as $\frac{2B}{1+B}$, where *B* is calculated as $\frac{\sum \left|x_{ij}-x_{ik}\right|}{\sum x_{ij}+x_{ik}}$, where *x*_*ij*_ is the presence of polymorphic locus *i* of individual *j*, while *x*_*ik*_ is the presence of polymorphic locus *i* of individual *k* [56, 57]. The value of *B* is between 0 (the loci with DNA fragments present are identical in both individuals) and 1 (the two individuals do not share any loci with DNA fragments present). The dissimilarity matrices were then analyzed using nonmetric multidimensional scaling (NMDS) [56] implemented in the R package “vegan” [57] with three dimensions, 1000 maximum number of random starts, and the stress ratio between two iterations exceeding 0.999999 in the search of a stable solution. NMDS is an ordination approach that finds those positions of individuals in reduced-dimension space that best reflects their AFLP dissimilarities. It is an iterative process using a steepest descent algorithm to minimize stress, the deviation between the final distance matrix to the original one. It is useful for recovering nonhierarchical structure while avoiding assumptions of the identity of individuals, normal data, or the underlying pattern of structure within the data [56, 58]. NMDS also provides a better fit to the data than may be obtained using other ordination techniques [59]. This procedure can visualize if a group of individuals collected from the same host web had similar AFLP profiles and determine whether the genetic information acquired from individuals collected in different host webs are distinctive. The goodness of fit of the NMDS model was measured using a stress value ranging from 0 to 1 (0 = excellent fit, 1 = poor fit).

#### Estimating the number of clusters that best fit the AFLP data using average silhouette approach

We utilized the average silhouette approach [60] to determine the optimal number of genetic partitions. First, we applied the binary dissimilarity matrices generated from AFLP phenotype tables to compute hierarchical clustering through different *C* values, where *C* is the number of clusters, ranging from 1 to the number of host webs we sampled. Specifically, the maximum *C* value (the total number of sampled host webs) for each species is: *A*. *miniaceus*: 13; *A*. cf. *fissifrons*: 14; *A*. *fasciatus*: 10; *N*. *trigonum*: 11). For each value of *C*, we calculated the silhouette score for each data point (the AFLP genetic profile per individual). The silhouette score is defined as (*b* − *a*) / *max* |(*a* − *b*)|, in which *a* is the mean intra-cluster distance and *b* is the mean nearest-cluster distance. The mean of silhouette scores of all data points is called the average silhouette score, which is a measure of how well samples are clustered with samples that are similar to themselves. Clustering models with a high average silhouette score are said to be “dense”, where data points in the same cluster are similar to each other, and “well separated” from where data points in different clusters are not very similar to each other. In other words, a high average silhouette score indicates a well-fit clustering, which balances the intra-clusters cohesion and inter-clusters separation [60]. This approach detects the optimal number of clusters, or genetic partitions, in an Argyrodinae species, in which the optimal number of clusters is the one that maximizes the average silhouette score through these iterations over a range of all *C* values [60]. The R scripts for NMDS and average silhouette approach can be found in S2 File.

#### Inference of population genetic structure using STRUCTURE and the estimation of relatedness

The genetic structure of the population was further examined using STRUCTURE v2.3.4 [61, 62] under a model assuming a haploid, admixture and independent allele frequencies. AFLPs are dominant markers in which band profiles are scored as presence/absence of PCR-amplified DNA fragments, such that the dominant homozygote (in which a DNA fragment is produced by both copies of the gene locus) cannot be distinguished from a heterozygote (in which a DNA fragment is produced by only one copy of the gene locus). Hence, we applied the haploid model in the STRUCTURE analyses. In addition, we assumed that admixture occurs among *Argyrodes* groups, i.e., kleptoparasites can have recent ancestors from multiple host webs as the distances between webs are relatively short and might not function as a geographical barrier for genetic structure. The assumption that the alleles are independent (i.e. linkage equilibrium) is applicable for AFLP markers as the restriction sites are randomly distributed in the genome and are unlikely to be closely associated. We tested the likelihood scores of the possible ancestries (the number *K*) from one to two more than the number of webs included in a population (e.g., we set a maximum *K* = 13 for the 11 webs of the *A*. *fasciatus* population that were included in the analysis). We performed this analysis with a burn-in period of 1,000,000 replications and a run length of 1,000,000 MCMC iterations for each *K*. The optimal number of *K* for each population of a species was estimated via the delta *K* statistics [62]. We also conducted the relatedness analyses [63] using the AFLP data from the groups that have multiple individuals in GenAlEx6.4 [64]. We tested if the probability of the relatedness within a group (individuals from the same web) is larger than the relatedness from randomly selected individuals in a population. This permutation-based method for relatedness analyses, bootstraps resampling to form 95% confidence intervals under the null hypothesis of no differentiation from a relatedness of 0 (unrelated), can only accommodate the webs with sample size larger than 2, as the 95% confidence intervals of the population means estimated from the samples will be the same to the permutation results when sample size equals to either 1 or 2.

#### Assessing geographic population differentiation using spatial autocorrelation

Spatial autocorrelation was used to compare the population genetic structure of group-living and solitary Argyrodinae. This procedure plots the autocorrelation coefficient ***r***, a measure of the correlation between the pairwise genetic similarity between pairs of individuals and the pairwise spatial distance between them. Calculation of genetic distance between pairs of individuals followed Huff et al [65]. Pairwise similarity of genetic distance and pairwise spatial distances were calculated in the Excel-based program GenAlEx6.4 [64]. Calculated values of ***r*** were plotted as a function of the specified distance classes. We did not allow empty distance classes (i.e., classes in which there were few or no pairs of individuals separated by that range of distances); as a result, we used different distance values for group-living *A*. *miniaceus* and *A*. cf. *fissifrons*; and for the solitary *A*. *fasciatus* and *N*. *trigonum*. Distances of 0-1m correspond to the group-living kleptoparasites in the same host web, because the host web is about 1 m in diameter. For each of the group-living species, we conducted separate analyses for different age/sex classes to determine at which stage these kleptoparasites were likely to disperse. We divided our samples of *A*. *miniaceus* into sub-adult and adult females, sub-adult and adult males, and juveniles, and individuals of *A*. cf. *fissifrons* into sub-adults and adults *versus* juveniles. Two statistical tests were performed to test the null hypothesis of no genetic structure: bootstrap estimates of ***r*** and a 95% confidence interval around the observed ***r*** in each distance class, and a permutation procedure that generates a distribution of ***r*** values under the assumption of no spatial structure (both described in detail in [66]). Within each distance class, 1000 bootstraps were performed by drawing pairwise distances (with replacement) from the set of pairwise distances in that distance class. We considered estimates of ***r*** for a particular distance class to be significant and biologically meaningful if both the estimated value of ***r*** and the 95% bootstrap confidence interval around the value of ***r*** fell outside the 95% confidence interval around ***r*** = 0.

## Results

**Fig 1:**
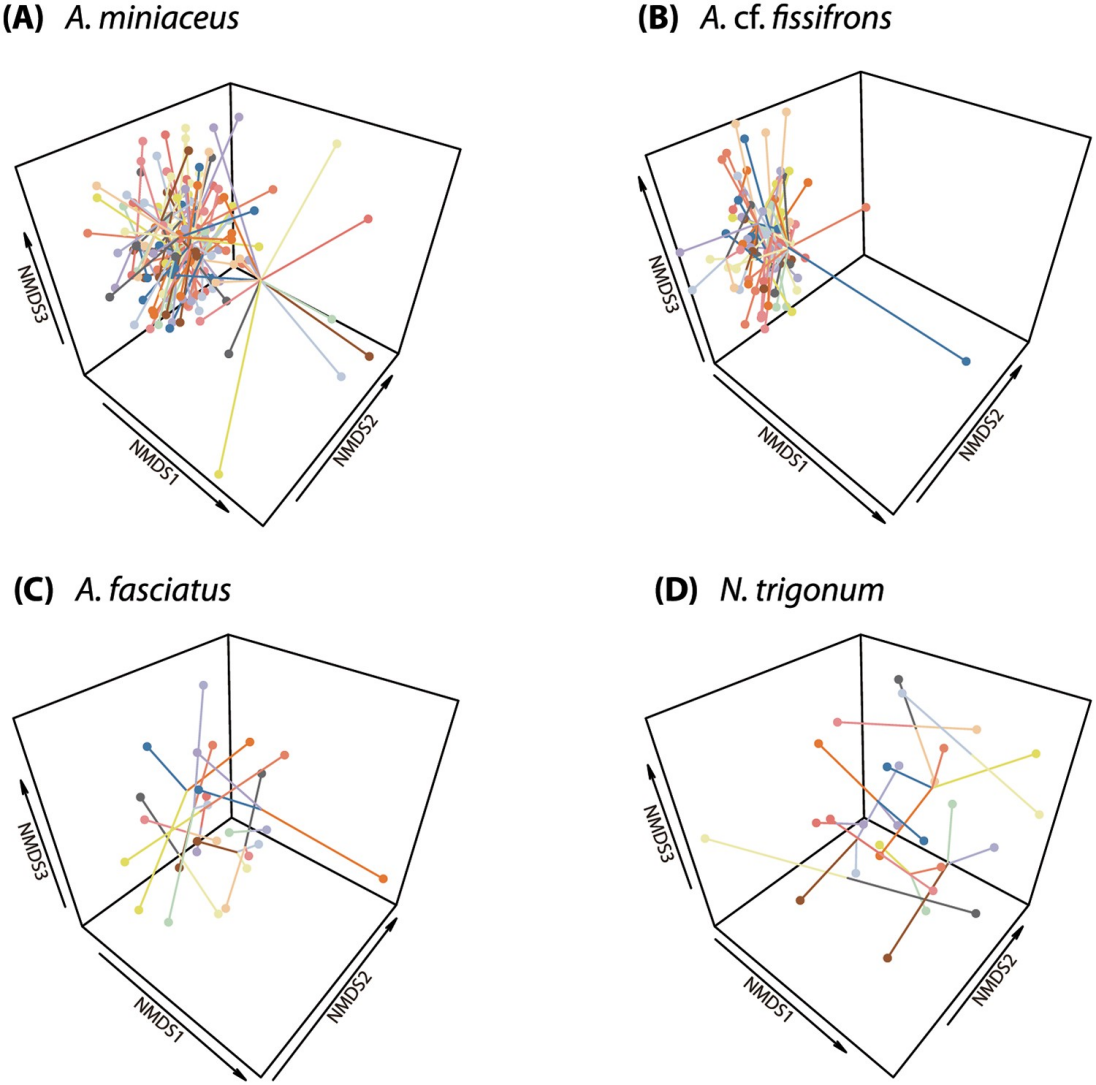
Three-dimensional nonmetric multidimensional scaling (NMDS) scatterplots. The NMDS scatterplots of AFLP dissimilarities of group-living species, (A) the group-living *A*. *miniaceus* and (B) the group-living *A*. cf. *fissifrons*, and (C) the solitary *A*. *fasciatus* and (D) the solitary *N*. *trigonum*. Data points are linked if they are collected from the same host web, while connected data points are shown in different colors if they are collected from different host webs.

Nonmetric multidimensional scaling (NMDS) analysis reached a solution at run 401 with a stress value of 0.186 for the group-living *A*. *miniaceus*, and at run 20 with a stress value of 0.105 for the group-living *A*. cf. *fissifrons*. The NMDS analysis of the solitary *A*. *fasciatus* reached a solution at run 20 with a stress values of 0.183, and at run 20 when the stress value was 0.020 for the solitary *N*. *trigonum*. These stress values are < 0.2, which indicates our NMDS representations are acceptable to AFLP binary markers [67] of the four Argyrodinae species. The NMDS analysis reveals that the genetic profiles of individuals in group-living species are less distant. In other words, there are more overlaps, as their AFLP dissimilarity clusters on NMDS plots were more densely connected to each point compared with solitary ones (Fig 1).

**Fig 2:**
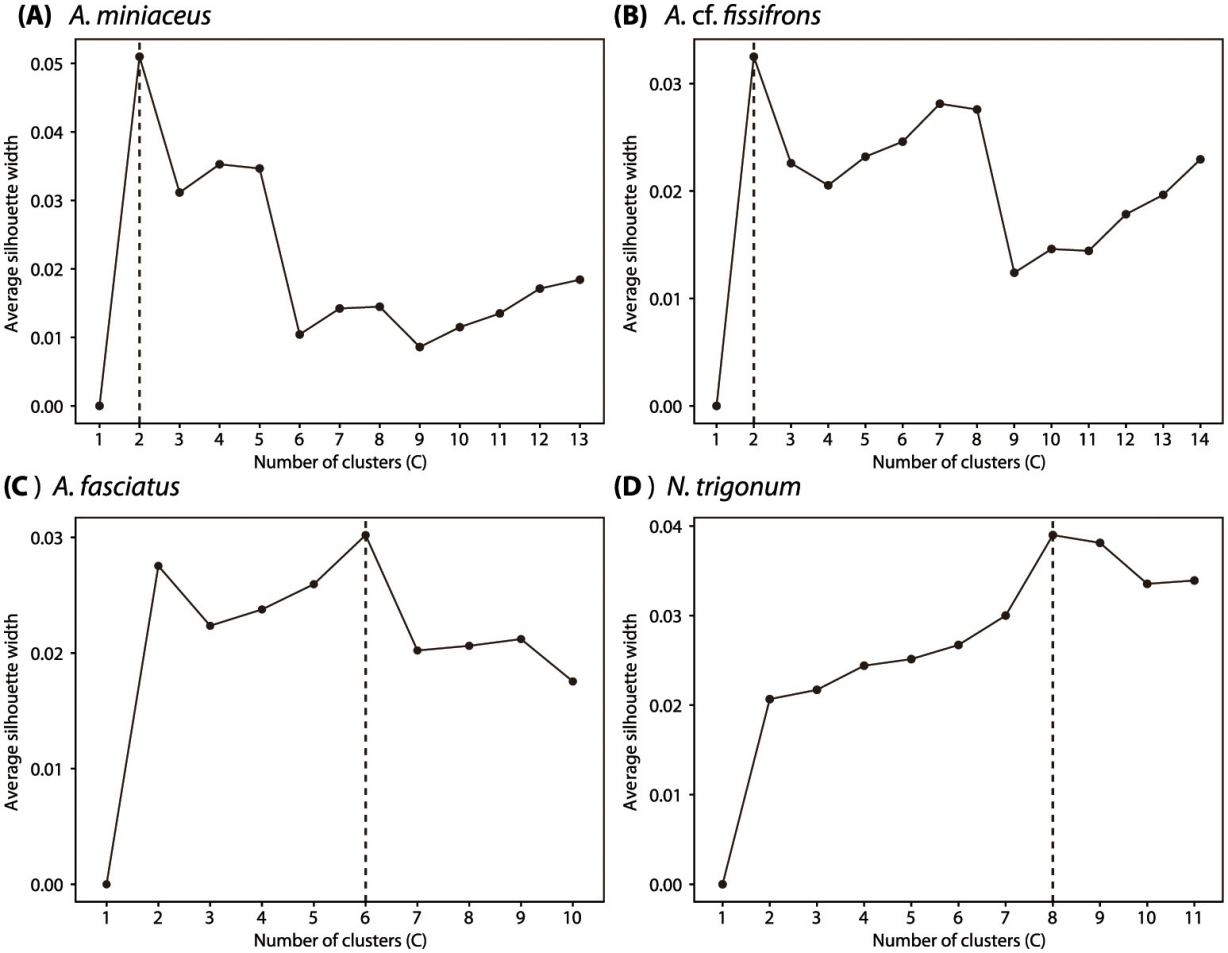
Estimated optimal number of clusters. The results of estimated optimal number of clusters using the average silhouette approach of group-living species, (A) *A*. *miniaceus* and (B) *A*. cf. *fissifrons*, and solitary species (C) *A*. *fasciatus* and (D) *N*. *trigonum*. A dashed line indicates an optimal number of genetic clusters.

An estimation of the optimal number of clusters using the average silhouette approach showed there are at least two genetic clusters in each of the two group-living species (*A*. *miniaceus*: *C* = 2; *A*. cf. *fissifrons*: *C* = 2). The average silhouette score calculations show that solitary Argyrodinae species have higher number of genetic clusters (*A*. *fasciatus*: *C* = 6; *N*. *trigonum*: *C* = 8) than group-living species (Fig 2). These results indicate that the boundary of the web, regardless of group-living or solitary species, could not fully explain the genetic subdivisions within populations. Rather, there are genetic clusters within populations of all species, while solitary species showed more genetic clusters and thus less structure in their populations.

**Fig 3:**
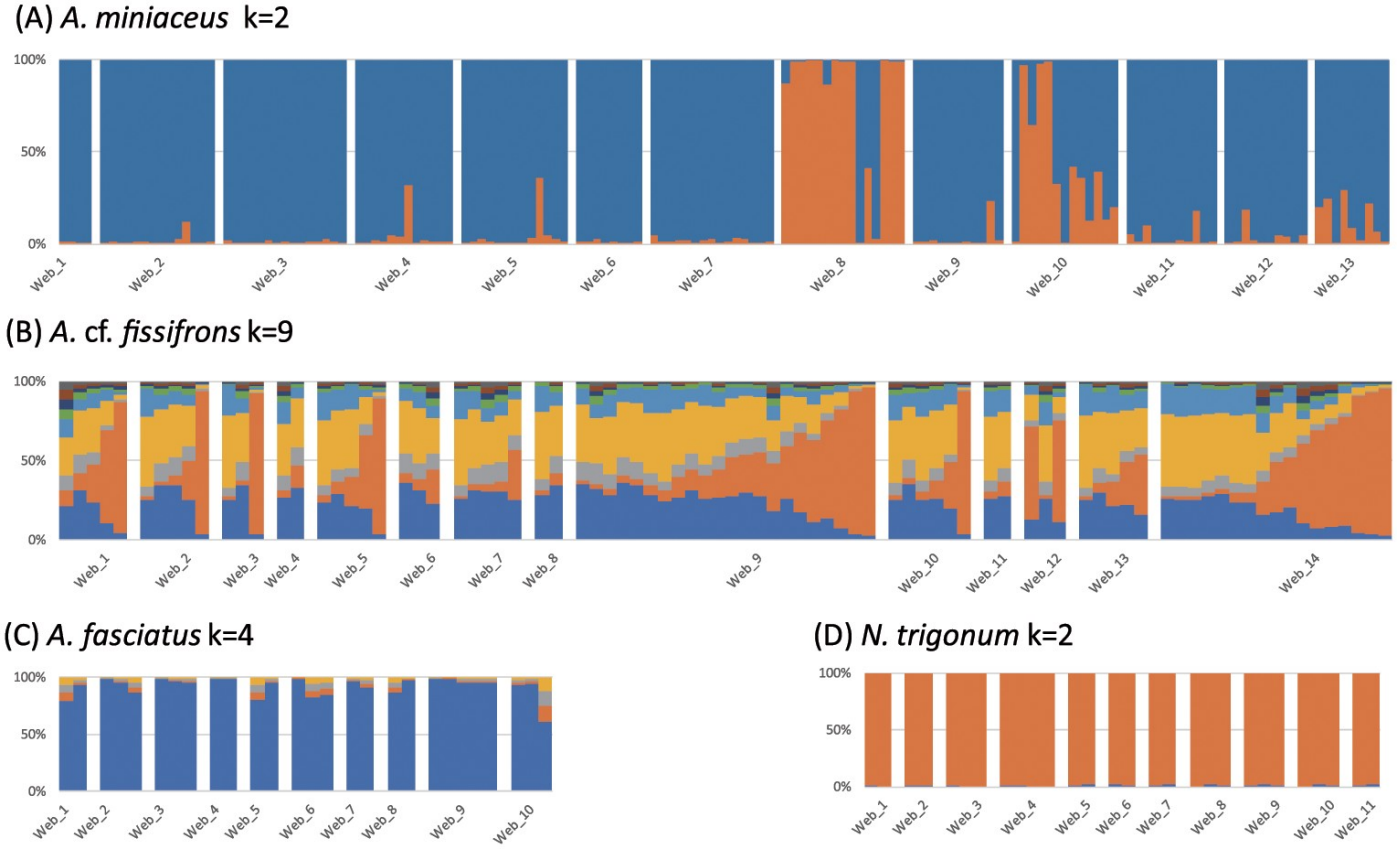
STRCTURE analyses. The optimal number of ancestries using the delta *K* approach and the corresponding results of STRUCTURE analyses shown as bar plots. Each colored segment is the likelihood of one individual assigned to a hypothetical population based on its AFLP genetic profile. Different colors are indicators of various ancestries: Proportions of (A) the population of the group-living *A*. *miniaceus* showed an optimal *K* = 2; (B) the population of the group-living *A*. cf. *fissifrons* showed optimal *K* = 9; and solitary species (C) *A*. *fasciatus* showed an optimal *K* = 4 and (D) *N*. *trigonum* showed *K* = 2.

The results of the STUCTURE analyses (Fig 3), which tested the optimal number of ancestries (*K*), revealed that the distribution of the optimal number of ancestries in the group-living *A*. *miniaceus* is consistently fixed (*K* = 2) among the individuals sampled within the same host web, and that this pattern is congruous across all groups collected from different host webs. The other group-living species, *A*. cf. *fissifrons*, showed a similar pattern but with more complicated ancestries (*K* = 9). The STRUCTURE results of the solitary *A*. *fasciatus* (*K* = 4) and *N*. *trigonum* (*K* = 2) showed a simpler pattern. While both solitary species revealed more than one ancestry in their populations, the proportion of ancestries of the individuals were dominated by a single ancestry.

**Fig 4:**
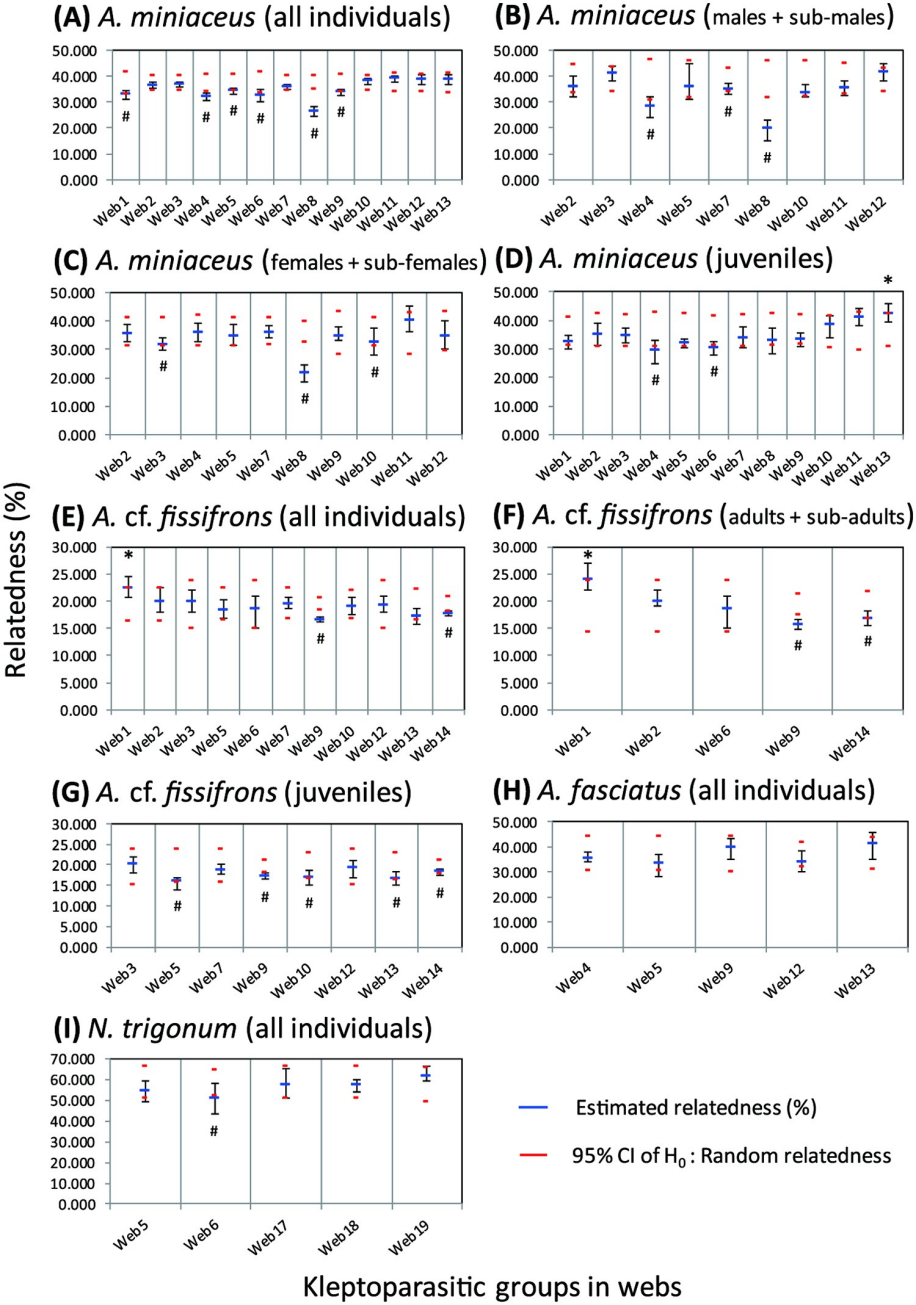
Estimated relatedness indices of each group collected from the same host web. Red dashed lines are upper and lower boundaries of 95% confidence intervals of the relatedness under the null hypothesis of random permutation. Blue dashes are the mean relatedness of each group collected from the same host web. Bars around blue dashes are the 95% confidence intervals of the estimated relatedness. * indicates that the mean relatedness of group members is significantly higher than random permutation. # indicates the mean relatedness of group members is significantly lower than random permutation. (A-D) the group-living *A*. *miniaceus*, in which (A) all individuals, (B) males, (C) females, and (D) juveniles; (E-G) the group-living *A*. cf. *fissifrons*, in which (E) all individuals, (F) adults, (G) juveniles; (H) all individuals of the solitary *A*. *fasciatus*; (I) All individuals of the solitary *N*. *trigonum*. The relatedness analyses only applied the groups where sample size larger than 2.

The AFLP genetic markers used to calculate the relatedness among group members collected from different host webs of the group-living *A*. *miniaceus*, showed that there was one group of juvenile members that had a significantly higher relatedness than random individuals (Fig 4; Web 13, *P* (mean-rand ≥ mean-data) = 0.031). There were six groups whose members had a lower relatedness than the randomly selected individuals in the population. In the group-living *A*. cf. *fissifrons*, there was one group whose members exhibited a significantly higher relatedness than randomly selected individuals from the population in the total of 14 webs (Fig 4; Web 1, *P* (mean-rand ≥ mean-data) = 0.036). In contrast, there were two groups that have lower relatedness than randomly selected individuals in the population (Fig 4).

**Fig 5:**
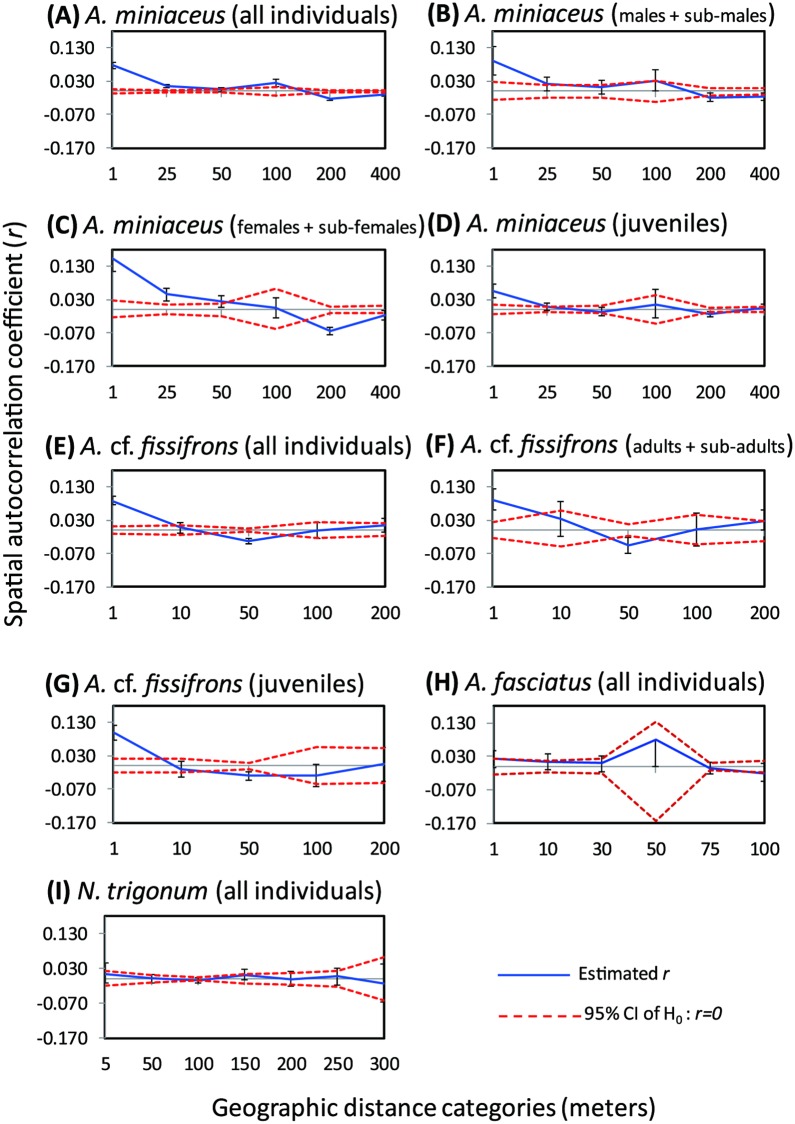
Spatial autocorrelation analyses of group-living species and solitary species. Red dashed lines = upper and lower boundaries of 95% confidence interval for values of the spatial autocorrelation coefficient, ***r***, under the null hypothesis of no geographic structure. Blue solid line connects ***r*** values for each distance category. Bars around computed ***r*** values are 95% bootstrap confidence intervals of null hypothesis ***r*** = 0. Analyses were performed for the group-living *A*. *miniaceus* (A)-(D), for (A) all individuals, (B) males + sub-males, (C) females + sub-females, and (D) juveniles; for the group-living *A*. cf. *fissifrons* (E)-(G), for (E) all individuals, (F) adults + sub-adults, and (G) juveniles; for all individuals of solitary *A*. *fasciatus* (H); for all individuals of the solitary *N*. *trigonum* (I).

Spatial autocorrelation between genetic and geographic distances showed that both group-living species have statistically significant spatial structures and have the highest ***r*** values at distances of 0–1 m, i.e., the distance corresponding to individuals in the same host web (***r*** = 0.077 and 0.088 for the group-living *A*. *miniaceus* and *A*. cf. *fissifrons*, respectively). In the *A*. *miniaceus* population, values of ***r*** were lower but still significant up to 25 m (Fig 5A). In *A*. cf. *fissifrons* no values of ***r*** were significantly higher at distances greater than 1 m (Fig 5E). In contrast, no autocorrelation between genetic and geographic distances were detected in either of the solitary species (Fig 5H and 5I).

Separate analyses for each developing stage and sex provide some insights into age-specific patterns of dispersal. The highest values of ***r*** for adult and sub-adult females, adult and sub-adult males, and juveniles of the group-living *A*. *miniaceus* are found at 0–1 m (Fig 5C). Values of ***r*** remain significantly high for sub-adult and adult males at 1 m. For sub-adult and adult females, the pattern at 25 is the same as that for the total sample (observed ***r*** values were greater than the upper limit of ***r*** expected under the null hypothesis of no population structure). No significant values of ***r*** were detected for juveniles at distances greater than 1 m (Fig 5B–5D). The only significant values of ***r*** for adult and sub-adult females, and juveniles of the group-living *A*. cf. *fissifrons* were at pairwise distances of 1 m or less—occupants of the same web (Fig 5F and 5G). In contrast with group-living species, there was no signal of age or sex-dependent population structure in either of the solitary species (Fig 5H and 5I).

## Discussion

Our DNA fingerprinting revealed greater genetic structure among group-living than solitary species of Argyrodinae spiders. NMDS analyses clearly identified more distinct clustering of individual AFLP profiles among group-living Argyrodinae species compared with solitary species (Fig 1). However, the low number of *A*. *fasciatus* samples (N = 27) might have caused a higher stress value (0.183) in the NMDS result, indicating that its cluster mapping might not be particularly accurate. These data suggest that long-distance exchanges of individuals are less frequent among the group-living species than in solitary species. While individuals of group-living species found on the same host web may or may not be closely related, individuals of solitary species are not closely related to individuals found on near-by host webs. The spatial autocorrelation analyses showed that genetic relatedness is correlated with geographic proximity in both of the group-living species, but not in the solitary species.

Previous studies have shown that resource size, i.e., higher prey availability and larger web size, is positively correlated with group size in group-living Argyrodinae [39, 40, 50, 51]. Thus, resource availability may have been an important ecological factor favoring initial group formation in the group-living Argyrodinae. If interactions among group members (related or non-related individuals) confer increased fitness, then persistence of aggregation over several generations would lead to increased relatedness among group members. In this way, kin selection could play a role in the evolution of Argyrodinae group-living systems after individuals had gathered into the host webs. However, long-term studies of inter- and intraspecific interactions of worldwide Argyrodinae species are required to corroborate or to falsify kinship components of kleptoparasitism in a larger sample of group-living and solitary species. In addition, the genetic basis of the transition from conspecific aggression to mutual tolerance among group members, which is essential for any group-living organism and especially for predatory species, remains to be investigated using modern genomic tools.

Cooperative kleptoparasitism has been observed in *A*. *miniaceus* [68] and *Faiditus globosus* [69] where the group members perform a host distracting behavior that apparently provides other group members with better access to the prey items caught in the host web. Our results indicate that cooperating individuals in groups of kleptoparasitic *A*. *miniaceus* or *A*. cf. *fissifrons* are likely to be interacting with non-kin, and in some cases, with kin individuals (Fig 4). Although a more pronounced genetic structure within populations of group-living than solitary species cannot simply be explained by web boundaries, the autocorrelation results show a higher than random genetic similarity within a short distance (*A*. *miniaceus* < 25 m and *A*. cf. *fissifrons* < 1 m, Fig 5). Thus, positive interactions among group members would occasionally lead to inclusive fitness benefits as well as direct fitness benefits. The observed variation in relatedness indicates that cooperating with kin is not obligate in either group-living species. In contrast, interactions between individuals of solitary kleptoparasite species, mainly involve courting, mating and, very occasionally, otherwise co-locating on the same web, none of which are likely to involve kin.

Differences in dispersal patterns and host web site tenacity may explain the differences in population structure of the two group-living species *A*. *miniaceus* and *A*. cf. *fissifrons*. Significant signals of autocorrelation between genetic and geographic distances were still detected at 25 m in *A*. *miniaceus*, while this signal was only apparent for individuals *A*. cf. *fissifrons* occupying the same host web (Fig 5A and 5E). The pattern of positive spatial autocorrelation in *A*. *miniaceus* differed between age classes, with significant signals at 25 m for sub-adult and adult females and within 1 m for juveniles (from hatchling to third instar) and males (Fig 5B to 5D). This genetic structure in *A*. *miniaceus* may reflect a combination of age-related dispersal patterns and the persistence of host webs. Host webs of *A*. *miniaceus* may not persist for long periods, and while juveniles disperse widely once they leave the natal web, most do not successfully enter and mature in other occupied webs. In contrast, later-dispersing females may be more successful at moving to and subsequently occupying nearby webs. Group-living *A*. cf. *fissifrons* specialize on the three-dimensional webs of *Cyrtophora*, which may persist in the same location for up to three months. As a result, *A*. cf. *fissifrons* are very likely to complete multiple generations in a single host web, so that the groups of these kleptoparasites would comprise extended families that occupy the host web until it is destroyed or abandoned by the host.

Clustered spacing patterns of hosts have been observed across many host-parasite combinations in Argyrodinae, including *Anelosimus eximius* hosting *F*. *ululans* [70], *N*. *clavipes* hosting *A*. *elevatus* and *A*. *caudatus*, and *Nephila clavipes* hosting *Argyrodes* spp. [71]. Usually, larger and more clustered host webs are occupied by more Argyrodinae than smaller or isolated host webs [72, 73], and among-web migration rates of group-living Argyrodinae are lower than that of the solitary species *N*. *trigonum*, the only solitary species that has been similarly studied [74]. Defense of the host web resource by group members and competition with intruders appear to be the reason for low between-web migration rates for group-living Argyrodinae [50]. The webs of host spiders are not permanent resource patches, and so the founder kleptoparasites in a host web may benefit from defending the web from intruders, reproducing and establishing a group before the host dies or moves its web.

Social groups of *A*. *miniaceus* (and possibly *A*. cf. *fissifrons*) are fundamentally different from “typical” cooperative spider species in an important way. Their host webs are a dynamic, frequently changing resource, because hosts may die or abandon a web and move to a new location. As a consequence, groups of these kleptoparasites can occupy a given host web for a few generations only; once the host web is destroyed or relocated, the members of the group either die or disperse in search of a new host web. Unlike individuals of cooperative species (e.g. *Anelosimus* (Theridiidae) or *Stegodyphus* (Eresidae) [19]) that construct their own webs, both juvenile and adult social kleptoparasites must retain the capacity for dispersal, which may limit opportunities to evolve the more intensely inbreeding social systems of *Anelosimus* and *Stegodyphus* [19]. The observation that group-living Argyrodinae such as *A*. *miniaceus* can cooperate with both kin and non-kin provides an interesting opportunity to investigate experimentally the role of both mutualism and inclusive fitness in the evolution of cooperative behavior in the same species.

The multiple independent evolutionary origins of group-living behaviors in Argyrodinae provide an excellent opportunity to investigate whether there is a role for pre-adapted sociality associated alleles [29]. However, the AFLP genetic data used in this study cannot identify which genes are associated with sociality in the group-living species. A genome-wide associated study (GWAS, [75]) that compares the populations of sister or closely related solitary/group-living species pairs is a more promising means of identifying candidates genes that are responsible for the social behavior in Argyrodinae species.

## Supporting information

S1 File Detailed methods of TE-AFLP DNA fingerprinting. (DOCX)

S2 File R script for NMDS and average silhouette analyses. (TXT)

S1 Data The raw binary matrix of AFLP markers of group-living *A*. *miniaceus*. (CSV)

S2 Data The raw binary matrix of AFLP markers of group-living *A*. cf. *fissifrons*. (CSV)

S3 Data The raw binary matrix of AFLP markers of solitary *A*. *fasciatus*. (CSV)

S4 Data The raw binary matrix of AFLP markers of solitary *N*. *trigonum*. (CSV)
