# Supplementary material for: Dual pathways in social evolution: Population genetic structure of group-living and solitary species of kleptoparasitic spiders (Argyrodinae: Theridiidae)
Source: PLoS One. 2018 Nov 29;13(11):e0208123. doi: 10.1371/journal.pone.0208123 (PMC6264510; doi:10.1371/journal.pone.0208123)
Supplement: S1 File — (DOCX) [file pone.0208123.s001.docx]

# Detailed methods of TE-AFLP DNA fingerprinting

We carried out DNA fingerprinting using the three-enzyme amplified fragment length polymorphism (TE-AFLP) method of van der Wurff et al. [1]. DNA fingerprints were collected for all individuals in one population of each species to examine population structure. We repeated fingerprints of 10 % of the individuals from each population to test for reliability and repeatability of fingerprints. We used three restriction enzymes, *Xba*I*, Bam*HI *and Rsa*I, to digest each DNA sample. The resulting DNA fragments were ligated to adaptors with sticky ends complementary to the *Xba*I and *Bam*H1 sticky ends of the DNA fragments. Each digestion/ligation reaction contained 1.0 μL of DNA extract, 2.0 μL of 10×ligase buffer, 2.0 μL of 500mM NaCl, 7.5 units ligase (NEB, USA), 1.25 units *Xba*I (Promega, USA), 6 units *Bam*HI (NEB, USA), 1 unit *Rsa*I (Promega, USA), 4.0 μL of *Bam*H1 adaptor (1 picoM/μL concentration), 4.0 μL of *Xba*1 adaptor (1 picoM/μL concentration), and enough water to make a 20 μL reaction (see van der Wurff et al. 2000 for sequence of the adaptors).

A subset of the DNA fragments was PCR amplified using primers complementary to the adaptors plus additional arbitrary bases; we used primer combination *Xba*I-CC and *Bam*HI-C. The *Xba*I-CC primer is complementary to one strand of the XbaI adaptor plus the two arbitrary bases “CC”, while the BamHI-C primer is complementary to one strand of the BamHI adaptor plus the arbitrary base “C” (see van der Wurff et al. 2000 for sequence of the primers). Each 12.75 μL PCR reaction contained 0.5 μL of DNA sample, 2.5 μL of 5×PCR buffer, 0.75 μL of 25mM MgCl_2_, 0.25 μL of *Bam*HI-C florescence-labeled primer (10pmol/ μL), 0.25 μL of *Xba*I-CC primer (10pmol/ μL), 0.125 μL of Taq polymerase (Gotaq, Promega, USA), and 0.25 μL of 10 mM dNTPs. We followed the thermal profile described by van der Wurff et al [1], which had 3 min denaturation at 95 °C, followed by 95 °C for 30 sec, 70 °C for 30 sec, and 72 °C for 60 sec for 10 cycles; 95 °C for 30 sec, 60 °C for 30 sec, and 72 °C for 60 sec for 40 cycles; and, finally, 72 °C for 20 min and stopping at 4°C. One microliter of PCR product was diluted with 29 ul of water before fragment sizing. The total number of individuals on which we attempted TE-AFLP fingerprinting and the number of individuals successfully fingerprinted are shown in Table 1.

**Fragment sizing** – The diluted PCR fragments were sized using a Beckman CEQ 8000 automatic sequencer and the resulting data were imported to the program Genemapper *v* 4.0 for scoring peak heights. The settings used in Genemapper *v* 4.0 were: fragment size range, 50bp to 600 bp; no normalization; common alleles deleted; and thresholds value type=absolute, and threshold value for inclusion in data set = 100.0 relative fluorescence units (rfu). After sizing the fragments in GeneMapper *v* 4.0, we collected the raw peak height data for each locus from all the individuals.

**Signal normalization** – We followed Whitlock *et al.* [2] to conduct peak height signal normalization and phenotype calling for each fragment. The raw peak height data that we screened in GeneMapper *v* 4.0 were imported in AFLPscore 1.4b [2], an interactive scripting program written in R. To normalize the raw peak height data, the sum of fluorescence intensity, *i* (in rfu), of every peak from each individual spider’s fingerprint is calculated. The program then calculates the median of fluorescence intensity, *m*, across the whole data table (individuals × loci). The ratio of *m*/*i* was used as the normalization factor and all the peak height values in the data table were multiplied by this normalization factor. This generates a new data table with normalized peak height values, corrected for individual reactions of different intensities.

**Testing reliability of data**- After normalizing the peak height of each locus in a species’ TE-AFLP fingerprints, we tested the mismatch rate between our formal data and repeated samples. Depending on the actual mean height of peaks in the data table for each species, we tried a series of peak height selection thresholds and locus selection thresholds to find the combination that generated the lowest mismatch rate for repeated data (i.e., two TE-AFLP fingerprints generated for the same individual). We aimed to retain the largest number of loci in our data matrices while still keeping the mismatch rate as low as possible and mismatched loci from the same individual were eliminated from the data matrix. Whitlock et al. [2] suggest that a mismatch rate below 3 to 4 % is adequate.

**Phenotype calling** - After finding the optimal combination of peak height selection threshold and locus selection threshold, we used this combination of selection thresholds to conduct phenotype calling, i.e., sort the peak heights of the loci in data matrices into “0” (peak absent) and “1” (peak present), and generate a phenotype table. Each peak, representing a piece of amplified DNA of a particular size, was considered to correspond to one particular gene locus. We eliminated from our data matrix any loci that showed a peak present in only one individual or showed a peak present in every individual except one (singletons). Because the TE-AFLP loci are scored as binary markers we refer to this as phenotypic data, to distinguish it from codominant markers such as microsatellites. The optimized phenotype tables were then used for the following analyses.

# Detailed Methods of spatial autocorrelation

Spatial autocorrelation was used to compare the population genetic structure of group-living and solitary Argyrodinae. This procedure plots the autocorrelation coefficient ***r***, a measure of pairwise genetic or phenotypic similarity between pairs of individuals, as a function of the pairwise spatial distance between them.

Pairwise phenotypic distance between individuals was calculated from the phenotype tables described above.

Calculation of phenotypic distance between pairs of individuals followed the method of Huff, Peakall (3), in which any loci that are in the same state in both individuals (i.e., 1 and 1 or 0 and 0) are given a value of 0, and any loci that differ in state between two individuals (i.e., 0 and 1, or 1 and 0) are given a value of 1. Pairwise phenotypic distance is the sum of scores across all loci. Pairwise geographic or spatial distance between individuals was calculated from the Universal Transverse Mercator positions calculated for each individual from the GPS coordinates recorded at the time of sample collection. Both pairwise similarity and pairwise spatial distances were calculated in the Excel-based program GenAlEx6.4 [4].

Spatial autocorrelation analyses were also performed in GenAlEx6.4. The autocorrelation coefficient, ***r***, can take values from -1 to +1, and is a measure of genetic similarity of pairs of individuals separated by specified linear distances [5]. Calculated values of ***r*** were plotted as a function of the specified distance classes (see Fig 5). Separate analyses were performed for each species, using the “single population” option, as we have data from only a single population of each species, and the “variable distance classes” option so that we could specify distance classes appropriate to the size of host webs, web clusters and collection transects. We did not allow empty distance classes (i.e., classes in which there were few or no pairs of individuals separated by that range of distances); as a result, we used different distance values for *A. miniaceus*, *A. kumadai* and the two solitary species. For the group living species *A. miniaceus* we used the distance classes 0 to1 m, >1 to 25 m, >25 to 50, >50 to 100 m, >100 to 200 m, and >200 to 400 m. Distances of 0-1m correspond to kleptoparasites in the same host web, because the host web is about 1 m in diameter. Individuals separated by distances >1m to 25 m represent individuals in neighboring webs in a cluster. The other distances compare individuals from more widely separated webs. For the group living species *A. kumadai*, we used the distance classes 0 to1 m, >1 to 10 m, >10 to 50, >50 to 100 m, and >100 to 200 m. For solitary species, we used distances as close to those used for group-living species as possible, while avoiding distance categories with few or no pair-wise distance samples.

For each of the group-living species we also did separate analyses of data from different age/sex classes to determine at which stage that these kleptoparasites were likely to disperse. We divided our samples of *A. miniaceus* into sub-adult and adult females, sub-adult and adult males, and juveniles; we subdivided our samples of *A. kumadai* into sub-adults and adults *versus* juveniles.

Two statistical tests were performed to test the null hypothesis of no genetic structure: bootstrap estimates of ***r*** that generate a 95% confidence interval around the observed estimate of ***r*** in each distance class, and a permutation procedure that generates a distribution of ***r*** values under the assumption of no spatial structure (both described in detail in [5]).

Within each distance class, 1000 bootstraps were performed by drawing pairwise distances (with replacement) from the set of pairwise distances in that distance class. The 1000 bootstrap estimates of ***r*** were ranked, and the 25th and 975^th^ values were used to define the 95% confidence interval around the observed estimate of ***r***. If the 95% bootstrap confidence interval around an estimate of ***r*** does not span zero, one can infer significant spatial structure.

The permutation procedure produces the upper and lower bounds of a 95% confidence interval around ***r*** = 0 at each distance class, ***r*** = 0 being the expected value under the assumption of no geographic structure. We used 1000 permutations in which pairwise phenotypic distances were randomly shuffled among spatial locations to generate the distribution of ***r*** under the hypothesis of no spatial structure, ranked the values of ***r*** obtained by permutations and used 25th and 975^th^ values to define the upper and lower boundaries of a 95% confidence interval around the hypothesis of no structure distances [5]. If a value of ***r*** estimated from the data falls outside of this range, then significant spatial structure can be inferred.

In this study, we considered estimates of ***r*** for a particular distance class to be significant and biologically meaningful if both the estimated value of ***r*** and the 95% bootstrap confidence interval around the value of ***r*** fell outside the 95% confidence interval around ***r*** = zero determined by permutation.

# References

1. van der Wurff AWG, Chan YL, van Straaien NM, Schouten J. TE-AFLP: combining rapidity and robustness in DNA fingerprinting. Nucleic Acids Res. 2000;28(24):e105. doi: 10.1093/nar/28.24.e105

2. Whitlock R, Hipperson H, Mannarelli M, Butlin R, Burke T. An objective, rapid and reproducible method for scoring AFLP peak height data that minimizes genotyping error. Mol Ecol Resour. 2008;8(4):725-735.

3. Huff DR, Peakall R, Smouse PE. RAPD variation within and among natural populations of outcrossing buffalograss [*Buchloe dactyloides* (Nutt.) Englm.] Theor Appl Genet. 1993;86(8):927-934. doi: 10.1007/BF00211043

4. Peakall R, Smouse PE GenAlEx 6.5: genetic analysis in Excel. Population genetic software for teaching and research—an update. Bioinformatics. 2012;28(19):2537-2539. doi: 10.1093/bioinformatics/bts460

5. Peakall R, Ruibal M, Lindenmayer DB. Spatial autocorrelation analysis offers new insights into gene flow in the Australian bush rat, *Rattus fuscipes*. Evolution. 2003;57(5):1182-1195.
